# Anisotropic Shrinkage Behavior of Overripe Papaya Slices (*Carica papaya* L. cv. Sunrise) during Convective Drying

**DOI:** 10.1021/acsomega.6c00261

**Published:** 2026-02-25

**Authors:** Giulliana Petean Torrano, Carmen Cecilia Tadini

**Affiliations:** Department of Chemical Engineering, Escola Politécnica, Universidade de São Paulo, Main Campus, 05508-010 São Paulo, São Paulo, Brazil; FoRC—Food Research Center, Universidade de São Paulo, Main Campus, 05508-010 São Paulo, São Paulo, Brazil

## Abstract

This study investigated the shrinkage behavior of overripe papaya slices during convective drying at 50 and 60 °C under 20% relative humidity, without disturbing the process. The Lewis model accurately described drying kinetics at both temperatures. Papaya slices exhibited anisotropic shrinkage, with area and height reductions of (15 ± 3) % and (76 ± 5) % at 50 °C and (26 ± 7) % and (87 ± 3) % at 60 °C, respectively. Although fundamental models were evaluated, only empirical correlations effectively described area and height shrinkage at both temperatures. At 50 °C, a deviation of approximately 10% from linearity was observed between water loss and sample volume reduction, while at 60 °C, a deviation below 5% occurred, suggesting an ideal shrinkage. These differences could be related to the gap between the sample and glass transition temperatures. Drying rate behavior was comparable at both temperatures, despite the occurrence of area shrinkage, which could be explained by the relatively small extent of area shrinkage. At 50 °C, no constant period was observed, followed by a single falling rate period, whereas at 60 °C, a short constant rate period was followed by two falling rate periods. Effective moisture diffusivity was evaluated with and without considering height shrinkage. When height shrinkage was neglected, diffusivity values were overestimated, particularly during the second falling period at 60 °C, when moisture mobility is expected to decrease. These findings highlight the importance of incorporating shrinkage into drying kinetics modeling.

## Graphical abstract

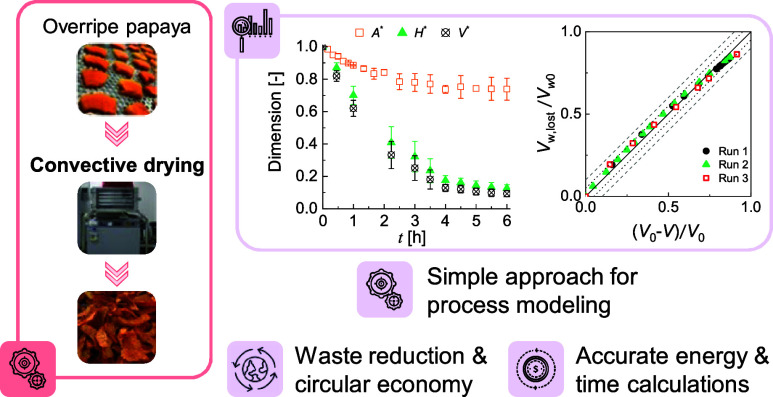

## 1 Introduction

Drying is one of the processes highly used in the food industry, primarily to improve food stability, and it is responsible for significant energy consumption. The most common method is convective drying, where hot dry air flows through the sample, responsible for mass and heat transfer.^1^ It is important to evaluate food behavior when subjected to drying, since changes in texture, matrix structure, or flavor could occur.^1,2^ Moreover, by evaluating moisture sorption isotherms, the relationship between sample moisture content and water activity can be understood, and drying time can be estimated. GAB and BET models are widely used for describing moisture sorption isotherms of foods, both for usually having a good fit for experimental data and for some thermodynamic information assigned to their parameters.^3^

When food is dried, one of the most important physical changes is the reduction of its volume. Heating and water removal generate stress within the cellular structure of the food matrix, resulting in the inability of the food tissue to hold its structural arrangement when the spaces occupied by water are emptied and air-filled. Then, the exterior skin structure collapsed, leading to changes in shape and a decrease in dimension.^1,4,5^ If shrinkage is not uniform, the formation of unbalanced stress can occur, leading to the failure of the material and surface cracking. Another phenomenon related to shrinkage is the decrease in the rehydration capability of the dried product.^1,4,5^ Shrinkage is affected by drying conditions and depends on the food matrix, as the structural rigidity of the cellular tissue and porosity influence the transport of intracellular and cell wall water, which strongly affects shrinkage.^4^ Therefore, shrinkage in food systems must be considered both for determining process conditions and for modeling the drying rate, since the latter is dependent on the area.

The shrinkage of the food matrix and its mathematical modeling have been studied. Mayor and Sereno^5^ categorized empirical and fundamental models, both linear and nonlinear, based on the reduced dimension: volume, area, or thickness. To monitor shrinkage during drying without disrupting the process, image processing can be used, providing a 2D measurement of the area. Recent studies have addressed this challenge in various ways: some used initial and final sample volumes to determine shrinkage;^6–8^ others calculated volume using true density and porosity^9^ or predicted volumetric deformation as a function of effective moisture diffusivity and density.^10^ Some studies monitored shrinkage throughout the drying process using scanner laser micrometers to follow two-dimensional changes,^11,12^ 2D imaging with suitable image processing,^13^ or an experimental device designed for in situ measurement of dimensions to calculate sample volume and area.^14^

Papaya (*Carica papaya* L.) is an important fruit crop grown widely in tropical and subtropical regions, with a world production of 14.7 million tons per year and Brazil as the fourth world producer (1.1 million tons/year) in 2024.^15^

Papaya fruit composition depends on cultivar, climatic conditions, soil, and harvesting time. Overall, it is composed of approximately 85–90 g of water per 100 g of papaya, rich in vitamins A and C, and has a high dietary fiber content, comprising approximately 1–2 g/100 g of its total weight.^16–19^ However, postharvest losses are greater than 40%, due to mechanical damage, overripening, or fungal diseases.^20–22^ Therefore, the drying of papaya can help minimize postharvest losses.

There are a few studies in the literature about sorption isotherms or drying of papaya pulp, and the majority of works focus on evaluating the impact of osmotic dehydration or osmotic and blanching pretreatment.^23–28^ However, the study of the sorption behavior and drying of papaya pulp allows the production of papaya powder. This powder can be a functional ingredient since papaya is rich in fiber, for example, pectin.

This work focuses on the convective drying, considering the shrinkage behavior, of papaya slices in a pilot-scale convective dryer under controlled conditions. This study allows the development of an efficient process for drying and producing pectin-rich papaya flour.

## 2 Materials and Methods

### 2.1 Raw Material Characterization and Selection

The unripe papaya fruits (*C. papaya* L. cv. Sunrise) were purchased from a local market in São Paulo, Brazil. To standardize the samples used in this work, the maturation of papayas was divided into six stages according to skin color and firmness analysis. For skin color, the stages were separated as follows: stage 0–100% green; stage 1–less than 15% yellow; stage 2–15% to 25% yellow; stage 3–25% to 50% yellow; stage 4–50% to 75% yellow; and stage 5—more than 75% yellow.^29^ The overripe papaya without seeds and peel was characterized concerning firmness, moisture content, water activity, pH, and soluble solid content.

#### 2.1.1 Firmness

The firmness of the pulp was measured by a penetration test in three points of the papaya fruit, without peel, using a texture analyzer (Stable Micro Systems, TA-XT2i, UK) with a cylindrical steel probe (P/6 model, 6 mm diameter) at a rate of 1.0 mm·s^–1^ and a penetration depth of 12 mm.

#### 2.1.2 Moisture Content

Overripe papayas at the target stage had their moisture content determined at 70 °C under vacuum pressure, according to method 934.06.^30^

#### 2.1.3 Water Activity

Water activity (*a*_w_) of papaya slices was determined by direct measurement at 25 °C (METER Group, AquaLab 3TE, USA).

#### 2.1.4 pH

The pH was measured by direct measurement at 20 °C (Mettler Toledo, FiveEasy F20, Switzerland).

#### 2.1.5 Soluble Solids Content (SS)

The soluble solids content (SS) was determined by direct measurement at 20 °C (Schmidt + Haensch, ATR, German) in the supernatant collected after centrifugation at 3214 g’s for 15 min (Eppendorf, Centrifuge 5804 R, USA).

### 2.2 Vapor Sorption Isotherms

Vapor sorption isotherms were obtained using a vapor sorption analyzer (Decagon Devices, VSA 1055, USA) at 25, 35, and 45 °C. Papaya pulp was cut into pieces and predried in a forced circulation oven (Nova Ética, N480, Brazil) until *a*_w_ = 0.91. Isotherms were run, in triplicate, under the dynamic method (DDI), at an *a*_w_ range from 0.9 to 0.1, and an airflow of 120 mL·min^–1^ for desorption. Four models were adjusted to isotherm experimental data: BET and modified BET,^31^ GAB,^32^ and double power,^33^ as follows, respectively

1 $$X_{w}=\frac{X_{mono}C_{BET}a_{w}}{\left(1-a_{w}\right)\left(1-a_{w}+C_{BET}a_{w}\right)}$$

2 $$X_{w}=\frac{X_{mono}C_{BET}a_{w}\left[1-\left(n+1\right){\left(a_{w}\right)}^{n}+n{\left(a_{w}\right)}^{n+1}\right]}{\left(1-a_{w}\right)\left[1-\left(C_{BET}-1\right)a_{w}-C_{BET}{\left(a_{w}\right)}^{n+1}\right]}$$

3 $$X_{w}=\frac{X_{mono}C_{GAB}Ka_{w}}{\left(1-Ka_{w}\right)\left(1-Ka_{w}+C_{GAB}Ka_{w}\right)}$$

4 $$X_{w}=k_{1}a_{w}^{n_{1}}+k_{2}a_{w}^{n_{2}}$$

wherein *X*_w_ is the water content [kg·kg^–1^ d.b.], *X*_mono_ is the monolayer water content [kg·kg^–1^ d.b.], *a*_w_ is the water activity in the food matrix [−], *C*_BET_ and *C*_GAB_ are model constants related to monolayer heat sorption [−], *n* is the number of layers adsorbed [−], *K* is the GAB correction factor [−], and *n*_1_, *n*_2_, *k*_1_, and *k*_2_ are the double power model constants [−]. For sigmoid isotherms (type II), *n*_1_ < 1 and *n*_2_ > 1, and for type III isotherms, *n*_1_ ≥ 1 and *n*_2_ > 1.^34^

#### 2.2.1 Thermodynamic Considerations

Theoretically, the BET and GAB models have thermodynamic information about the average heat of sorption associated with the monolayer (*H*_m_), multilayer (*H*_n_), and heat of condensation of pure water (λ), according to eqs 5–8 ^3,35,36^

5 $$C_{BET}\approx C_{0,BET}\exp\left(\frac{\Delta H_{C_{BET}}}{RT}\right);,\Delta H_{C_{BET}}=H_{m}-\lambda$$

6 $$C_{GAB}\approx C_{0,GAB}\exp\left(\frac{\Delta H_{C_{GAB}}}{RT}\right);,\Delta H_{C_{GAB}}=H_{m}-H_{n}$$

7 $$K\approx K_{0}\exp\left(\frac{\Delta H_{K}}{RT}\right);,\Delta H_{K}=\lambda -H_{n}$$

8 $$X_{mono}\approx X_{0,mono}\exp\left(\frac{q_{m}}{RT}\right)$$

wherein *R* is the gas constant [J·mol^–1^·K^–1^], *T* is the temperature [K], *C*_0,BET_ [−], *C*_0,GAB_ [−], *K*_0_ [−], *X*_0,mono_ [kg·kg^–1^ d.b.], and *q*_m_ [J·mol^–1^] are adjustable constants for the temperature effect, and Δ*H*_*C*_BET__[J·mol^–1^], Δ*H*_*C*_GAB__[J·mol^–1^], and Δ*H*_K_ [J·mol^–1^] are functions of heat of water sorption.^35^

It is possible to predict these model constants for temperatures beyond those used in isotherms by plotting the linearized form of eqs 5–8.

### 2.3 Convective Drying

Drying experiments were conducted in a convective drying oven (Labmaq, LM-ES20, Brazil) equipped with removable trays, each placed on a scale for real-time weight measurement, with controlled temperature (*T*) and relative humidity (*RH*). The oven has a quartz window on the roof, allowing the acquisition of images of a single sample during the experiment using an S6D stereomicroscope (Leica, Germany).

The papaya pulp was cut lengthwise and then into slices with 6 mm thickness, (3.6 ± 0.4) cm of length, (1.8 ± 0.4) cm of width, and an area-to-volume ratio of 0.17. The slices were placed on the top tray, with each batch containing between 80 and 100 papaya slices (320 to 400 g). The drying conditions studied were *RH* = 20%, air speed of 4 m·s^–1^ and *T* = (50 and 60) °C, and the slices were dried until constant weight. The *RH* and *T* were controlled by the convective drying oven and had an oscillation of 0.5% and 1 °C, respectively, during the process. The weight of the tray was measured each minute by the scale with real-time weight measurement. Experiments were conducted in triplicate.

To describe drying kinetics, five semitheoretical models were adjusted to experimental data, as shown in Table 1. The moisture ratio (*MR*) can be described as

9 $$MR=\frac{X_{w}-X_{eq}}{X_{w0}-X_{eq}}$$

wherein *X*_w0_ is the initial water content [kg·kg^–1^ d.b.] and *X*_eq_ is the water content in equilibrium [kg·kg^–1^ d.b.], estimated from vapor sorption isotherms.

**Table 1:** Models Used in This Work to Describe the Drying Kinetics

model	equation	references
Lewis	MR = exp(−*k*_1_ *t*)	^37^
extension of Lewis	$MR=\exp\left(-k_{1}t-{\left(k_{2}t\right)}^{2}\right)$	^38^
page	MR = exp(−*k*_1_ *t* ^*n*^)	^39,40^
modified page	$MR=\exp{\left(-k_{1}t\right)}^{n}$	^39–41^
Henderson–Pabis	MR = α exp(−*k*_1_ *t*)	^39,40^

*MR* is the moisture ratio [−], *t* is the time [s], and *k*_1_ [s^–1^], *k*_2_ [s^–1^], *n*, and α are model constants [−].

#### 2.3.1 Shrinkage Behavior

To evaluate shrinkage, images of the slices were taken throughout the drying experiments without disturbance, and the area was determined using image processing software (LAS6; Leica, Switzerland). In separate experiments under the same conditions, the papaya slice height was measured every 30 min, in triplicate, using a micrometer (Mitutoyo, model 103–137, Brazil).

In this work, sample dimensions and water content were used in the normalized form to minimize deviations caused by the heterogeneous matrix of papaya slices. The dimensionless water content (*X*_w_*) was expressed as

10 $$X_{w}^{*}=\frac{X_{w}}{X_{w0}}$$

The dimensionless area (A*), height (H*), and volume (V*) of the sample can be determined by

11 $$A^{*}=\frac{A}{A_{0}};,H^{*}=\frac{H}{H_{0}}$$

12 $$V^{*}=\frac{V}{V_{0}}=\frac{AH}{A_{0}H_{0}}$$

wherein *V*, *A*, and *H* are the volume [m^3^], area [m^2^], and height [m] of the sample, and the subscript 0 indicates the initial value. The volume was considered as *A* × *H*, without defining a specific geometry, since the area was determined by the image processing software, and the height was directly measured.

In the drying of real food, shrinkage is neither three-dimensional nor unidimensional.^42^ As a first approach, the “vertical model” considers a unidimensional shrinkage, where the area shrinkage is negligible compared to the height one. The three-dimensional approaches, which are empirical or fundamental models, can be divided into three groups based on model consideration: the isotropic approach, consisting of linear shrinkage behavior throughout the whole drying experiment; some deviations of this linear behavior; and porosity variation.^5,43,44^ Therefore, the area and height shrinkage behaviors were evaluated according to the models shown in Table 2.

**Table 2:** Models Used in This Work to Describe the Shrinkage Behavior^a^

model	equations	references
vertical	$H_{vert}^{*}=\frac{V}{V_{0}};\;A_{vert}^{*}=1$	^5^
isotropic	$H_{iso}^{*}=A_{iso}^{*}={\left(\frac{V}{V_{0}}\right)}^{1/3}$	^5^
combined	$$\begin{array}{l} H_{comb}^{*}={\left(H_{iso}^{*}\right)}^{b}{\left(H_{vert}^{*}\right)}^{\left(1-b\right)};\\ A_{comb}^{*}={\left(A_{iso}^{*}\right)}^{b};b=\frac{1}{2}\left(1-X_{w}^{*}\right) \end{array}$$	^37^
linear fundamental	$\frac{V}{V_{0}}=\left(\frac{X_{w}+0.8}{X_{w0}+0.8}\right);H^{*}={\left(\frac{V}{V_{0}}\right)}^{2/3}$	^5^
empirical:		
linear	H* = *d* + *eX*_w_*; A* = *d* + *eX*_w_*	^43,44^
linear with apparent density	$$\begin{array}{l} \frac{A_{t}}{A_{0}}={\left[aX_{wt}+b\right]}^{n}\\ a=\frac{\rho_{0}}{\left(X_{w0}+1\right)};b=1+a-\rho_{0} \end{array}$$	^45^
quadratic	$H^{*}=f{X_{w}^{*}}^{2}+gX_{w}^{*}+h$	^5^
power	H* = *iexp* (*jX*_w_*)	^5^

a *a, b, d, e, f*, *h*, *g*, *i*, and *j* are adjustable constants [−] and ρ_0_ is the apparent density [kg**·**m^–3^].

The dimensionless volume of lost water (*V*_w,lost_*) and volume lost by the sample (*V*_s,lost_*) were calculated according to

13 $$V_{w,lost}^{*}=\frac{V_{w,removed}}{V_{w0}};V_{s,lost}^{*}=\frac{V_{0}-V}{V_{0}}$$

wherein *V*_w,removed_ is the volume of removed water [m^3^], *V* is the volume of the sample [m^3^], and the subscript 0 indicates the initial value.

#### 2.3.2 Drying Rate

The drying rate was calculated according to

14 $$Ṙ=-\frac{dX_{w}}{dt}\frac{m_{s}}{A\left(t\right)}$$

wherein *X*_w_ is the water content [kg·kg^–1^ d.b.], *t* is the time [s], *m*_*s*_ is the dry mass [kg], and *A*(*t*) is the area [m^2^] as a function of time.^1^

For each falling period found in the drying rate versus time curves, the effective moisture diffusivity (*D*_eff_) was obtained by nonlinear regression according to the solution of Fick’s law proposed by Crank^46^

15 $$\frac{X_{w}-X_{eq}}{X_{w0}-X_{eq}}=MR=\sum_{i=0}^{\infty }\frac{8}{{\left(2i+1\right)}^{2}+\pi^{2}}\exp\left\{-{\left(2i+1\right)}^{2}\frac{\pi^{2}D_{eff}t}{4L^{2}}\right\}$$

wherein *X*_w_ is the water content [kg·kg^–1^ d.b.], *X*_eq_ is the water content in equilibrium [kg·kg^–1^ d.b.], *t* is the time [s], *D*_eff_ is the effective moisture diffusivity [m^2^·s^–1^], and *L* is the characteristic length [m].

### 2.4 Statistical Analyses

Data analyses were performed using Origin 2024 and Statgraphics Centurion XV. Significant differences were determined using analysis of variance and the Tukey test for multiple ranges with a significance level of *p* < 0.05. Model selection was made according to the *R*_adj_^2^ and the RMSE. A residual analysis was made, and 95% confidence intervals were used.

## 3 Results and Discussion

### 3.1 Raw Material Characterization and Selection

In order to target waste reduction, the ripening stage of papayas used in this work was stage 5. Firmness analysis was introduced to select fruits instead of using only skin color as a parameter. Changes in skin color, firmness, and *SS* of samples along the maturation are shown in Figure 1. The pH did not change significantly (*p* > 0.05) along with the maturation, varying from (5.35 ± 0.03) to (5.50 ± 0.03).

**Figure 1:**
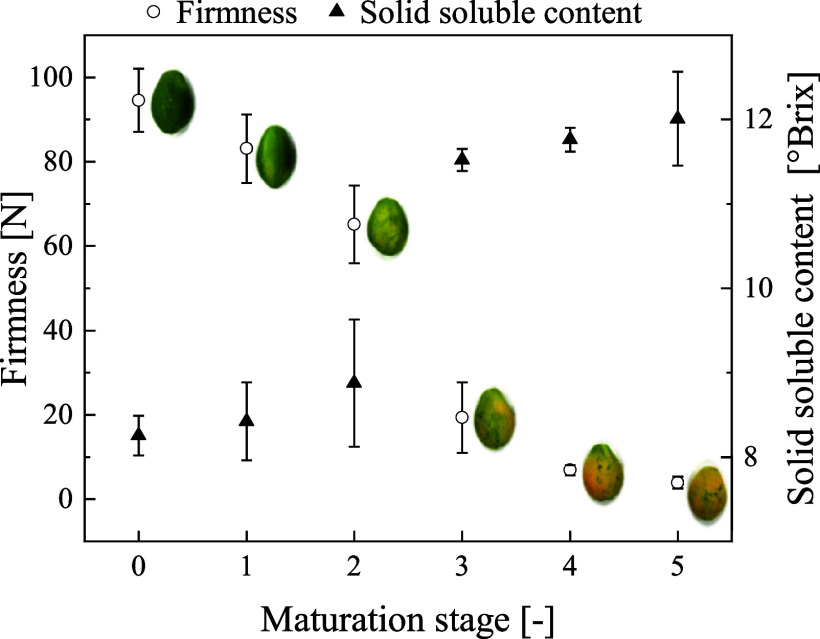
Firmness and solid soluble content of papaya pulp in each maturation stage.

As the firmness parameter for stage 5 was defined (3.9 ± 1.4) N, all samples had their firmness evaluated before being used in this work. The initial values of raw papayas were *X*_w0_ of (6.2 ± 0.5) kg water·kg^–1^ d.b. and *a*_w_ of 0.988 ± 0.001.

### 3.2 Vapor Sorption Isotherms

The vapor sorption isotherms for papaya slices (Figure 2) can be classified as type III isotherms, which are typically found in foods with high-soluble-sugar content.^47^ The models adjusted to isotherms in this work can be divided into two groups: with and without the monolayer hypothesis. As discussed by Peleg^34^ and Roos,^3^ BET and GAB are the most common models used in the literature because of their good fit to experimental data, even as the monolayer hypothesis is controversial in applications on food matrices, and these models do not consider changes in the food matrix, such as shrinkage or glass transition. From the four models adjusted (Table 3), only the double power model proposed by Peleg^33^ does not consider the monolayer hypothesis, and it is focused on the sigmoidal shape of isotherms, having a good fit for types II and III.

**Figure 2:**
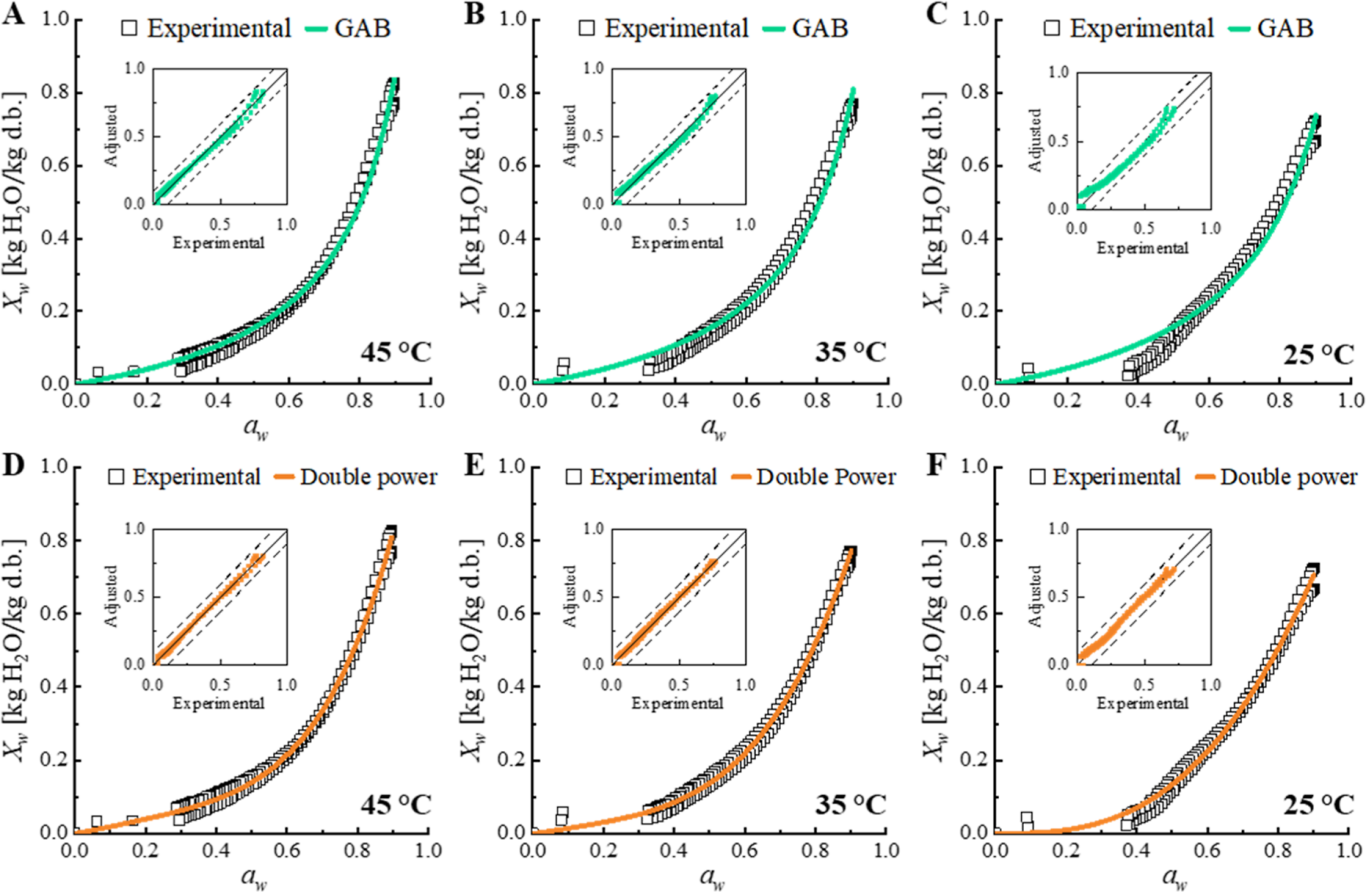
Vapor desorption isotherms of papaya slices obtained at 45 °C (A,D), 35 °C (B,E), and 25 °C (C,F). Experimental values, adjusted GAB, and double power model and their respective parity charts.

**Table 3:** Model Parameters Adjusted to Experimental Data of Papaya Slices Desorption Curves at 25, 35, and 45 °C

model	*T* [°C]	parameters				*R*_adj_^2^	RMSE [kg·kg^–1^ d.b.]
		*X*_mono_ [kg·kg^–1^ d.b.]	*C*	*K*	*n*		
BET	45	0.096	4.19			0.964	0.0451
	35	0.089	7.01			0.943	0.0556
	25	0.082	18.4			0.895	0.0718
modified BET	45	0.152	0.88		15.5	0.995	0.0158
	35	0.151	0.77		13.2	0.981	0.0324
	25	0.154	0.85		12.0	0.982	0.0294
GAB	45	0.171	1.10	0.923		0.990	0.0235
	35	0.181	1.08	0.904		0.986	0.0272
	25	0.198	1.06	0.873		0.971	0.0379
		***k***_**1**_	***k***_**2**_	***n***_**1**_	***n***_**2**_		
double power	45	0.206	1.05	1.03	4.76	0.995	0.0162
	35	0.905	0.20	4.07	1.28	0.995	0.0157
	25	0.351	0.60	2.83	2.83	0.992	0.0192

In this work, GAB, the double power, and modified BET models showed a good fit to the experimental data in desorption isotherms at 25, 35, and 45 °C (Table 3 and Figure 2).

The double power model was the best for describing the data (highest *R*_adj_^2^, lowest RMSE). However, the GAB model was selected because it offers two advantages: it allows comparing data with the literature, as it is one of the most commonly used models to describe isotherms, and it enables the prediction of model constants for temperatures beyond those used in isotherms, as described in Section 2.2.1. Kurozawa et al.^48^ studied the water desorption in papaya using saturated salt solutions at (40, 50, 60, and 70) °C and reported that the GAB model was the best fit for their experimental data. The parameters obtained in this work are different from those found by the cited authors, with higher *X*_mono_ and lower *C*_GAB_ and *K*. Kurozawa et al.^48^ reported *K* values higher than 1, even though they should be lower than 1. As this upper limit was established for *K* in this work, this could explain the divergences observed in the parameter values found. In addition, *C*_GAB_ < 2 results in an upward concave isotherm, instead of a sigmoid shape,^3^ which is consistent with the shape of the isotherms and with the parameter values obtained in this work.

Since convective drying was conducted at higher temperatures than the isotherms (50 and 60 °C), these model parameters were predicted for these temperatures to allow the determination of *X*_eq_. Values for these model parameters, *R* ^*2*^, RMSE, and *X*_eq_, considering *a*_w_ = 0.2, are shown in the Supporting Information (Table S1).

### 3.3 Convective Drying

The *X*_w_ of samples decreased from (6.7 ± 0.1) kg water·kg^–1^ d.b. to (0.05 ± 0.01) kg water·kg^–1^ d.b. at 50 °C and to (0.03 ± 0.01) kg water·kg^–1^ d.b. at 60 °C, whereas the *a*_w_ of samples decreased from (0.989 ± 0.001) to (0.402 ± 0.002) at 50 °C and to (0.384 ± 0.002) at 60 °C.

Applying the *X*_eq_ obtained by the isotherms (Section 3.2), the *MR* can be calculated and the aforementioned models (Section 2.3) for drying kinetics were adjusted. All models showed a good fit to the experimental data, with an *R*_adj_^2^ higher than 0.979 and an RMSE lower than 0.038 (Table 4).

**Table 4:** Model Parameters Adjusted to Experimental Data of Papaya Slices for Drying Experiments at 50 and 60 °C and 20% RH

model	*T* [°C]	parameters				*R*_adj_^2^	RMSE
		*k*_1_ [10^–4^ s^–1^]	*k*_2_ [10^–5^ s^–1^]	*n* [−]	α [−]		
Lewis	50	1.34				0.995	0.018
	60	1.87				0.979	0.038
extension of Lewis	50	1.42	0			0.992	0.022
	60	1.67	5.09			0.981	0.036
Page	50	1.37		1.00		0.994	0.019
	60	0.99		1.07		0.981	0.036
modified Page	50	1.36		0.94		0.996	0.015
	60	1.84		1.08		0.981	0.036
Henderson–Pabis	50	1.29			0.96	0.996	0.015
	60	1.92			1.03	0.980	0.037

The models used are equivalent to the Lewis model when *k*_2_ = 0 for the extension of the Lewis model, *n* = 1 for the Page and modified Page models, and α = 1 for the Henderson–Pabis model. These values were observed for the Page and extension of Lewis models at 50 °C, and similar values were obtained for the Page, modified Page, and Henderson–Pabis models at 60 °C. Therefore, the Lewis model was chosen to describe the drying kinetics at both 50 and 60 °C (Figure 3), as it has the fewest parameters, which avoids overestimation while providing a good fit.

**Figure 3:**
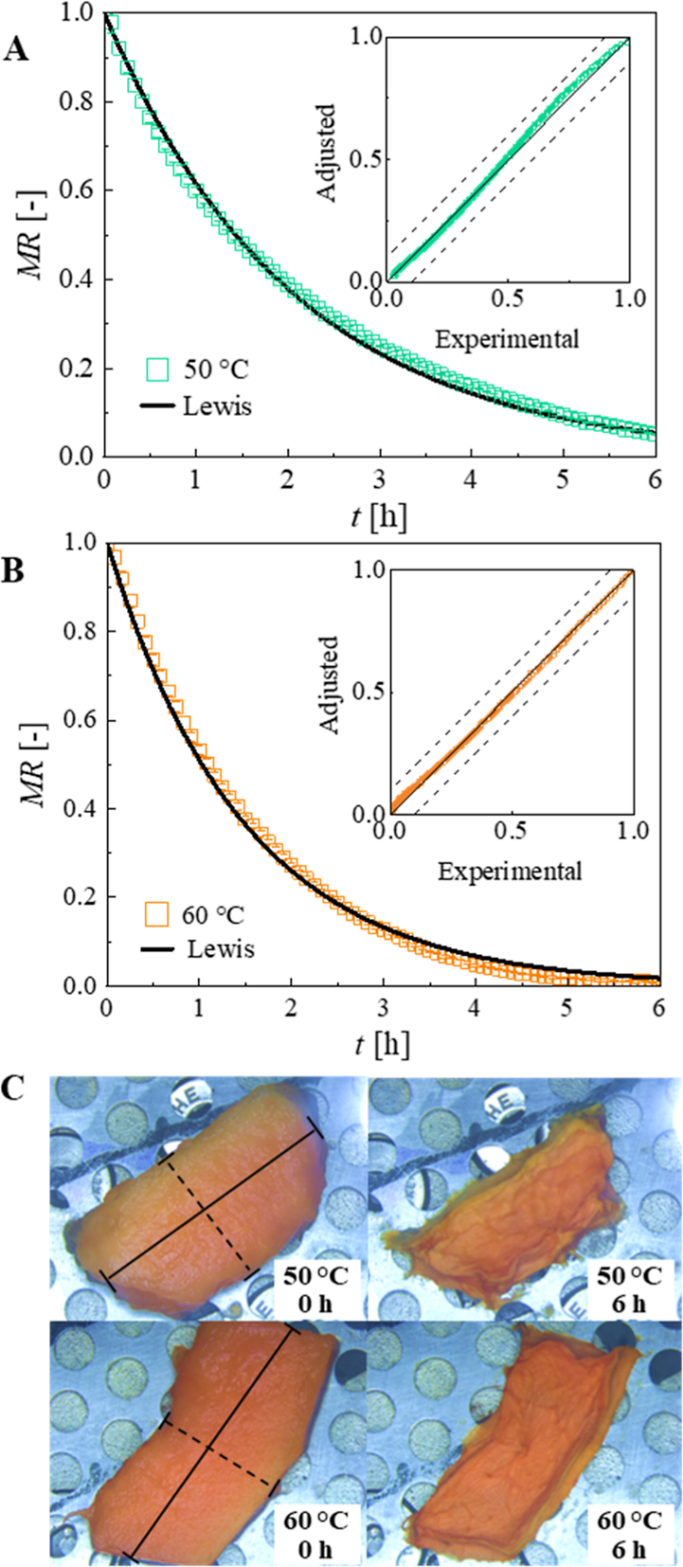
Experimental values of *MR* of papaya slices in function of drying time, adjusted Lewis model, and their respective parity charts at 50 °C, 20% *RH* (A) and 60 °C, 20% *RH* (B). Photos of the slices before and after the drying process (C), where the solid line indicates the length and the dashed line indicates the width measurements.

#### 3.3.1 Shrinkage Behavior

The area influences the drying rate, which makes it necessary to evaluate the shrinkage behavior. Changes in area (A*), height (H*), and volume (V*) of samples throughout drying are shown in Figure 4A–D as a function of time and dimensionless moisture content for 50 and 60 °C at 20% of *RH*.

**Figure 4:**
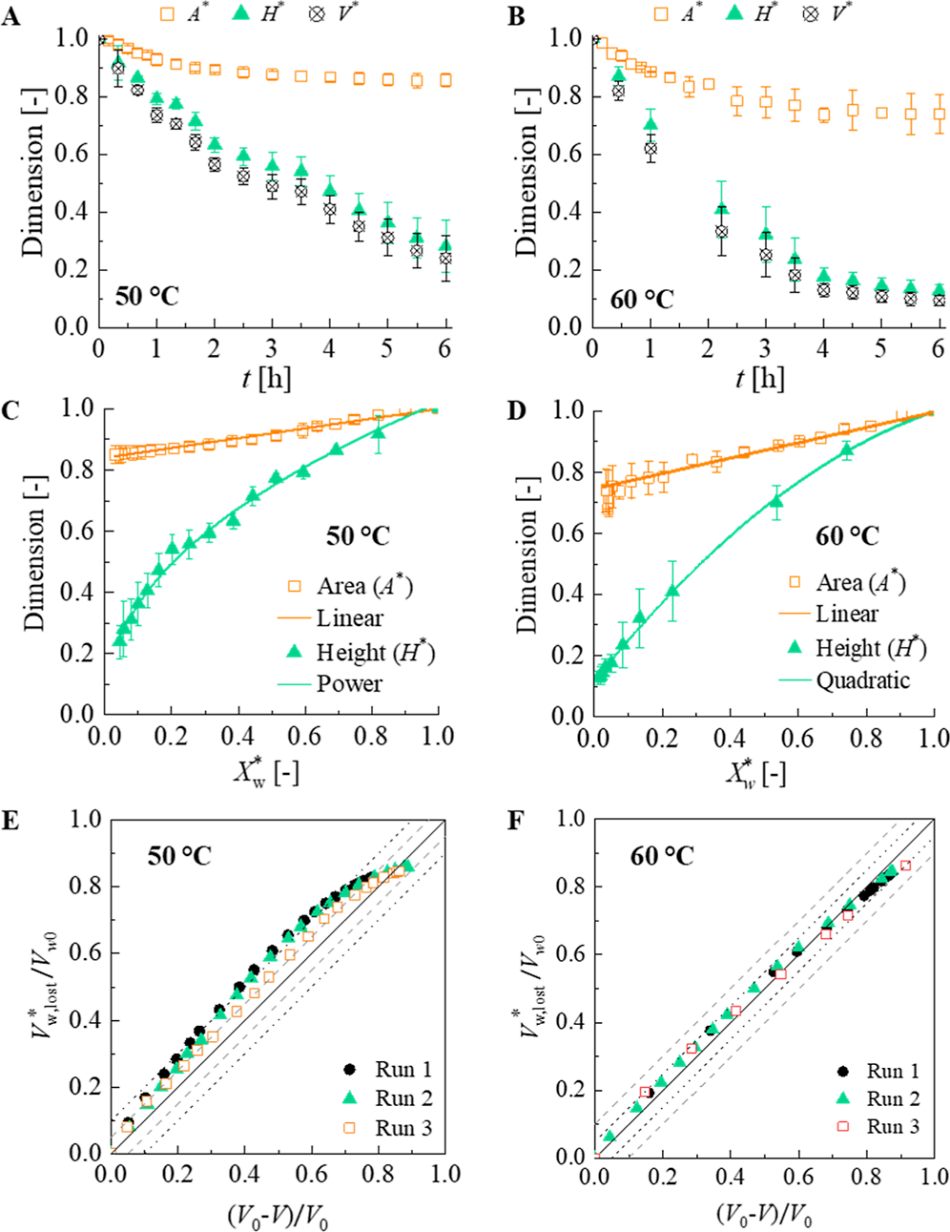
Dimensionless area (A*), height (H*), and volume (V*) of papaya slices throughout drying as a function of time and dimensionless moisture content at 50 °C (A and C, respectively) and 60 °C (B and D, respectively); dimensionless volume of lost water as a function of the dimensionless volume lost of the sample for papaya slices dried at 50 °C, 20% *RH* (E) and 60 °C, 20% *RH* (F). The solid line indicates the linear behavior, which corresponds to an ideal shrinkage. The dashed line and the dotted line indicate a 10% and 5% deviation, respectively, from the linear behavior.

The shrinkage behavior differed between dimensions at both temperatures. At 50 °C, A* shrinkage ceased after 2.5 h of drying, while the H* shrinkage continued throughout drying. Conversely, at 60 °C, shrinkage in both dimensions ceased before the end of drying, stabilizing after 2.5 h for A* and after 4.5 h for H*.

Furthermore, the extension of shrinkage at the end of drying differed, with final values of (0.85 ± 0.03) for A* and (0.24 ± 0.05) for H* at 50 °C, 20% *RH*, and (0.74 ± 0.07) for A* and (0.13 ± 0.03) for H* at 60 °C, 20% *RH*. This indicated that the shrinkage of papaya slices exhibited anisotropic behavior that neither the vertical nor the isotropic models can accurately describe. Therefore, the other models (Table 2) were adjusted to experimental data. Empirical correlations showed a good fit: a linear relationship for area (A*) shrinkage both at 50 and 60 °C, a power law for height (H*) shrinkage at 50 °C, and a quadratic relationship for height (H*) shrinkage at 60 °C (Figure 4C,D). The parity charts for each model are available in the Supporting Information (Figure S1).

Typically, uniform and pronounced shrinkage occurs at low temperatures due to the flat moisture transportation pattern, whereas higher temperatures often lead to lower shrinkage due to the case hardening effect.^4^ However, in the present study, the shrinkage at 60 °C was greater than at 50 °C.

For area shrinkage, a similar behavior was observed at both temperatures: at the beginning of drying, the surface has a high moisture content and is in a rubbery state, resulting in a linear relationship between area shrinkage and time, which persisted until 2.5 h of drying. For height shrinkage, the behavior differed between temperatures, and shrinkage ceased only at 60 °C after 5 h of drying. This difference may be related to the glass transition, which is influenced by the moisture content and solid fraction.

At the beginning of drying, the difference between the sample temperature (*T*_p_) and the glass transition temperature (*T*_g_) is great in both cases, resulting in high-rate shrinkage. As the drying process occurs, this difference decreases, reducing the extension of the shrinkage rate. When a greater reduction in moisture content occurs, the glass transition temperature increases, and the drying temperature might become lower than the glass transition temperature. This could lead to a transition from a rubbery to a glassy state, reducing the mobility of the solid matrix and consequently the rate and extension of shrinkage. Kurozawa et al.^49^ reported that *T*_p_ at 70 °C was above *T*_g_ throughout the process, while at 40 °C and moisture content below 0.2 kg·kg^-1^ d.b., a glass transition was observed, which resulted in a greater extension of shrinkage for 70 °C than 40 °C. This result is similar to what was found in this study.

The relationship between the dimensionless volume of lost water (*V*_w,lost_*) and dimensionless volume lost by the sample (*V*_s,lost_*) was evaluated at 50 and 60 °C (Figure 4E,F). At 50 °C, the volume of water removed was greater than the volume reduction, and nonlinear behavior was observed throughout the drying process, with a deviation from linear behavior close to 10% for most of the process. At 60 °C, a linear behavior occurred, with a deviation of less than 5%. Thus, at 60 °C, the *V*_w,lost_ increased proportionally with *V*_*s*,lost_, which is considered an ideal shrinkage and is related to the rubbery state of the sample.^4,49^ The behavior observed at 50 and 60 °C was similar to what was reported by Kurozawa et al.^49^ at 40 and 70 °C, respectively.

#### 3.3.2 Drying Rate

The drying rate ( Ṙ ) is plotted as a function of time and dimensionless moisture content (Figure 5), at 50 and 60 °C, considering both constant area ( ${Ṙ}_{A_{0}}$ ) and area shrinkage ( ${Ṙ}_{A\left(t\right)}$ ).

**Figure 5:**
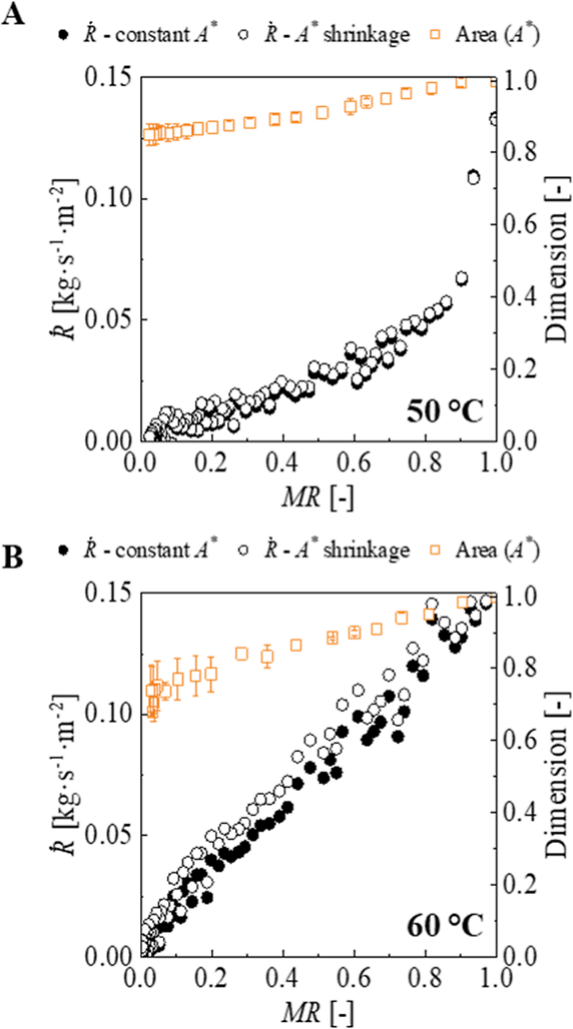
Drying rates and area of papaya slices as a function of *MR* at 50 °C, 20% *RH* (A) and 60 °C, 20% *RH* (B). Drying rate values assuming constant area and area shrinkage.

For ${Ṙ}_{A\left(t\right)}$ at 50 °C, no constant drying rate period was observed, and just one falling rate period occurred. In contrast, at 60 °C, a short constant drying rate period was observed for *X*_w_* between 0.97 and 0.8, followed by a falling rate period. A second falling rate period was observed when *X*_w_* was below 0.15, occurring after 3 h of drying and corresponding to the end of area shrinkage. At both temperatures, similar behavior was observed for ${Ṙ}_{A_{0}}$ and ${Ṙ}_{A\left(t\right)}$ . However, lower drying rate values were obtained throughout the drying.

When the actual area is included in the drying rate calculation, depending on the shrinkage behavior and the porosity of the solid matrix, a significant constant drying rate period might be expected. This phenomenon has been reported for potato^13^ and yacón,^14^ but not for apple.^13^ In the present study, the drying rate tendencies remained similar, and the values obtained were comparable, despite the area correction at both temperatures. This could be explained by the relatively small extent of area shrinkage observed (15% at 50 °C and 26% at 60 °C).

Gutiérrez et al.^43^ investigated the drying kinetics of papaya (*C. papaya* L. “Red Maradol”) at 50, 60, and 70 °C and air velocities of 1.0, 1.5, and 2.0 m·s^–1^. The cross-sectional area and depth of the sample holder were 45 mm × 75 mm and 5 mm, respectively. They reported the absence of a constant drying rate period and only one falling period across all temperatures. They calculated the drying rate by incorporating area shrinkage, which was considered as a linear function of dimensionless moisture content. Furthermore, they found that variations in air velocity had a slight impact on the drying process. These findings differ slightly from the present study, where a short constant drying rate period and a second falling rate at 60 °C were observed. These differences could be attributed to the higher air velocity used (4 m·s^–1^), which they indicated influenced the drying process, and the *RH*, which was not specified in their study. Another potential influencing factor is the difference in food matrix, as they used the “Red Maradol” cultivar, while this study used the “Sunrise” cultivar.

The effective diffusivity is calculated according to eq 15 for the one falling period at 50 °C and the two falling periods at 60 °C, separately. The first 4 terms of the series were used since no change in effective diffusivity was observed by adding more terms. To take the shrinkage phenomenon into consideration, the decreasing values of height were applied to determine effective diffusivity. At 50 °C, without considering the shrinkage, the *D*_eff_ obtained was 3.735 × 10^–10^ m^2^·s^–1^, and when considering the shrinkage, the value was reduced to 1.366 × 10^–10^ m^2^·s^–1^. At 60 °C, the same was observed, with lower values both for the first and second falling period when considering shrinkage: 3.889 × 10^–10^ and 5.274 × 10^–10^ m^2^·s^–1^ without shrinkage, and 1.812 × 10^–10^ and 0.863 × 10^–10^ m^2^·s^–1^ considering shrinkage. Values obtained at 60 °C were higher than at 50 °C, as expected, since a higher temperature leads to higher diffusivity. At 60 °C, without considering the shrinkage, the *D*_eff_ obtained for the second falling period is higher than that obtained for the first falling period, which is not in accordance with the experimental data, since at the end of the drying process, the drying rate and the diffusion are reduced. This corroborates the importance of including the shrinkage in the *D*_eff_ calculation. The values obtained in this study are similar to what was reported by Kurozawa et al.^49^ for papaya cubes dried at 40 and 70 °C, considering shrinkage, although they do not present the drying rate of the process or any discussion about the falling rate period.

## 4 Conclusions

This study investigated the drying and shrinkage behavior of overripe papaya slices in a pilot-scale convective dryer. Vapor sorption isotherms obtained at 25, 35, and 45 °C were classified as type III, and the GAB model was selected to describe the experimental data. As for drying, the Lewis model was chosen to describe drying kinetics at both temperatures.

Papaya slices exhibited anisotropic shrinkage: at 50 °C and 20% *RH*, area and height reduced by (15 ± 3) % and (76 ± 5) %, respectively, and at 60 °C and 20% *RH*, by (26 ± 7) % and (87 ± 3) %. Area shrinkage ceased after 2.5 h of drying at both temperatures, while height shrinkage continued throughout drying at 50 °C and stabilized after 4.5 h at 60 °C. Although fundamental models were evaluated, only empirical correlations adequately described the shrinkage behavior: a linear relationship for area shrinkage at both temperatures, a power law for height shrinkage at 50 °C, and a quadratic relationship for height shrinkage at 60 °C.

At 50 °C, the relationship between the volume of removed water and volume lost by the sample throughout the drying process was nonlinear, with a deviation of approximately 10% from linear behavior for most of the process. In contrast, at 60 °C, a deviation of less than 5% occurred, suggesting an ideal shrinkage. This difference can be attributed to the difference between the sample and glass transition temperatures.

The drying rate was evaluated under two assumptions: constant area and area shrinkage. At 50 °C, no constant rate period was observed, followed by a one-falling-rate period. Conversely, at 60 °C, a short constant drying rate period was observed, followed by two falling rate periods. The drying rate values obtained were comparable, despite the area correction at both temperatures. This could be explained by the relatively small extent of area shrinkage observed (15% at 50 °C and 26% at 60 °C). Effective diffusivity was calculated for each falling rate period, with and without the height shrinkage. Without height shrinkage, diffusivity was overestimated at both temperatures, and the value for the second falling period at 60 °C was higher than the one for the first falling period, contradicting expected behavior as diffusivity usually decreases at the end of drying. These findings highlight the importance of incorporating shrinkage behavior into drying models.

Therefore, this work provides a simple approach for studying shrinkage and drying phenomena in food matrices, contributing to improved energy efficiency. Moreover, it is aligned with circular economy principles by using agro-industrial waste as raw material, supporting the waste reduction and recycling targets of Sustainable Development Goal 12.

## Supplementary Material



## Notes

Predicted model parameters for the temperatures used in convective drying; values for model parameters, *R* ^2^, RMSE, and *X*_eq_, considering *a*_w_ = 0.2; parity charts for area and height shrinkage models at 50 °C, 20% RH (A) and 60 °C, 20% RH (B) (PDF)

## Nomenclature

*A*: area of the sample [m^2^]
*A**: dimensionless area of the sample [−]
*A*_0_: initial area of the sample [m^2^]
*A*_comb_*: dimensionless area of the sample, combined model [−]
*A*_iso_*: dimensionless area of the sample, isotropic model [−]
*A*_vert_*: dimensionless area of the sample, vertical model [−]
*a*_w_: water activity [−]
*C*_0,BET_: adjustable constant for the temperature effect [−]
*C*_BET_: model constant [−]
*C*_0,GAB_: adjustable constant for the temperature effect [−]
*C*_GAB_: model constant [−]
*D*_eff_: effective moisture diffusivity [m^2^·s^–1^]
*H*: height of the sample [m]
*H**: dimensionless height of the sample [−]
*H*_comb_*: dimensionless height of the sample, combined model [−]
*H*_iso_*: dimensionless height of the sample, isotropic model [−]
*H*_vert_*: dimensionless height of the sample, vertical model [−]
*H*_0_: initial height of the sample [m]
*H*_m_: average heat of sorption associated with the monolayer [J·mol^–1^]
*H*_n_: average heat of sorption associated with the multilayer [J·mol^–1^]
*K*: GAB correction factor [−]
*K*_0_: adjustable constant for the temperature effect [−]
*k*_1_: model constant [−]
*k*_2_: model constant [−]
*L*: characteristic length [m]
MR: moisture ratio [−]
*m*_s_: dry mass of the sample [kg]
*n*: model constant [−]
*n*_1_: model constant [−]
*n*_2_: model constant [−]
*q*_m_: adjustable constant for the temperature effect [J·mol^–1^]
*R*: gas constant [J·mol^–1^·K^–1^]
Ṙ: drying rate [kg·m^–2^·s^–1^]
RH: relative humidity [%]
SS: soluble solids content [°Brix]
*T*: temperature [K]
*T*_p_: sample temperature [K]
*T*_g_: glass transition temperature [K]
*t*: time [s]
*V*: volume of the sample [m^3^]
*V**: dimensionless volume of the sample [−]
*V*_w,lost_*: dimensionless volume of lost water [−]
*V*_s,lost_*: dimensionless volume lost by the sample [−]
*V*_0_: initial volume of the sample [m^3^]
*V*_w_: volume of water [m^3^]
*V*_w0_: initial volume of water [m^3^]
*V*_w,removed_: volume of removed water [m^3^]
*X*_eq_: water content in equilibrium [kg·kg^–1^ d.b.]
*X*_mono_: monolayer water content [kg·kg^–1^ d.b.]
*X*_0,mono_: adjustable constant for the temperature effect [kg·kg^–1^ d.b.]
*X*_*w*_: water content [kg·kg^–1^ d.b.]
*X**_w_: dimensionless water content [−]
*X*_w0_: initial water content [kg·kg^–1^ d.b.]

## Greek Letters

α: model constant [−]
Δ*H*_*C*_BET__: model constant related to monolayer and heat of water sorption [J·mol^–1^]
Δ*H*_*C*_GAB__: model constant related to monolayer, multilayer, and heat of water sorption [J·mol^–1^]
Δ*H*_K_: model constant related to multilayer and the heat of condensation of pure water [J·mol^–1^]
λ: heat of condensation of pure water [J·mol^–1^]
ρ_0_: apparent density [kg·m^–3^]
