# Supplementary material for: Anisotropic Shrinkage Behavior of Overripe Papaya Slices (Carica papaya L. cv. Sunrise) during Convective Drying
Source: ACS Omega. 2026 Feb 25;11(9):15523–32. doi: 10.1021/acsomega.6c00261 (PMC12980162; doi:10.1021/acsomega.6c00261)
Supplement: Supplementary file 1 [file ao6c00261_si_001.pdf]

Anisotropic shrinkage behavior of overripe papaya slices (*Carica papaya* L. cv. Sunrise) during convective drying

Giulliana Petean Torrano and Carmen Cecilia Tadini[*]

Universidade de São Paulo, Escola Politécnica, Department of Chemical Engineering, Main *Campus*, 05508-010, São Paulo, SP, Brazil

FoRC – Food Research Center, Universidade de São Paulo

*Corresponding author.

Name: Carmen Cecilia Tadini

E-mail address: catadini@usp.br

Table S1. Predicted model parameters for (50 and 60) ºC and their respective X_{eq}, obtained from the linearized form of equations (5–8).

T	**Predicted parameters**			X_{eq} **[kg·kg⁻¹ d.b.]**
**[°C]**	X_{mono} **[kg·kg⁻¹ d.b.]**	***C***	***K***	
50	0.166	1.11	0.937	0.041
60	0.154	1.13	0.961	0.040
R^2	0.993	0.992	0.986	
RMSE	0.009	0.002	0.005	

$\Delta H_{C_{GAB}}$ = -1.54 [kJ·mol^{-1}]; ΔH_K = -2.22 [kJ·mol^{-1}]; $H_m = 2.26\times10^3$ [kJ·mol^{-1}]; $H_n = 2.26\times10^3$ [kJ·mol^{-1}]; q_m = 5.76 [kJ·mol^{-1}]

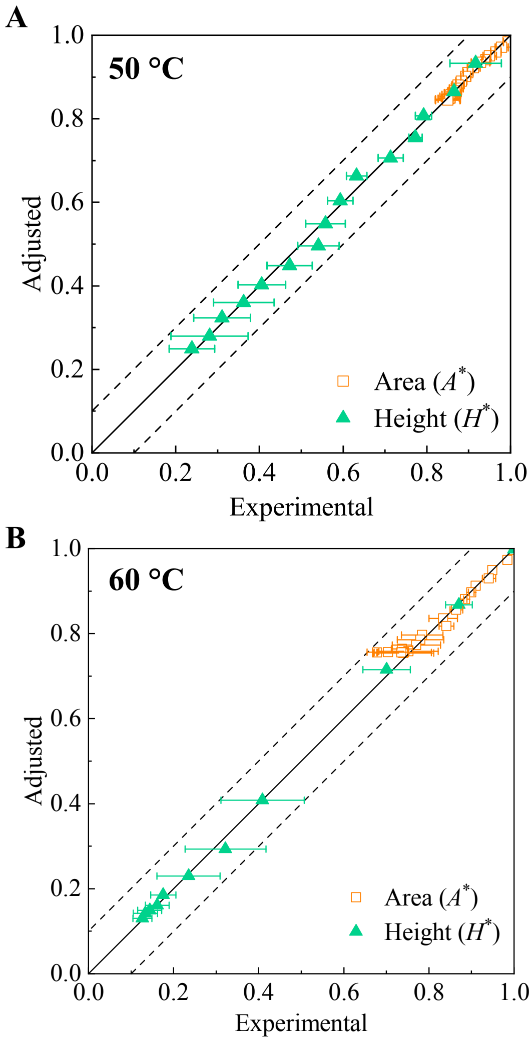

Figure S1. Parity charts for an empirical linear correlation for dimensionless area (A^*) shrinkage at 50°C (A) and 60 °C (B), an empirical power law correlation for dimensionless height (H^*) shrinkage at 50 °C (A), and an empirical quadratic correlation for dimensionless height (H^*) shrinkage at 60 °C (B).
